# Supplementary material for: Emergent patterns from probabilistic generalizations of lateral activation and inhibition
Source: J R Soc Interface. 2016 May;13(118):20151077. doi: 10.1098/rsif.2015.1077 (PMC4892256; doi:10.1098/rsif.2015.1077)
Supplement: Supplementary Material [file rsif20151077supp1.pdf]

Emergent Patterns from Probabilistic Generalisations of Lateral Activation and Inhibition
Supplementary Material

L. Willis[1], A. Kabla[2]

[1] *The Sainsbury Laboratory, University of Cambridge, Bateman Street, Cambridge CB2 1LR, UK*

[2] *Department of Engineering, University of Cambridge, Trumpington Street, Cambridge CB2 1PZ, UK*

1 A discrete and probabilistic model of lateral activation and inhibition: the corresponding time-continuous version

The continuous-time Markov chain $U(t)$ evolves the configuration of colours over a spatial domain Ω that is discretized as a square lattice; at any time t, a configuration assigns to each discrete lattice site $\mathbf{x}$ in Ω (each *cell*) one of two colours, either *white* $U_{\mathbf{x}}(t) = W$, or *black* $U_{\mathbf{x}}(t) = B$.

At time $t = 0$, the cells have colours that are independent and identically distributed, taking value W with probability 1/2. This constitutes the *initial condition*, the configuration $U(0)$, which ensures that initially the colours of different cells are uncoupled. For the cells $\mathbf{x}$ *outside* Ω, their colours are prescribed at time $t = 0$ and thereafter remain fixed for all $t > 0$; these permanently coloured cells constitute the *boundary condition.* The colour prescriptions for different $\mathbf{x}$s outside of Ω are independent and identically distributed, taking value W with probability 1/2. Thereafter $U(t)$ evolves as $\mathbf{x}$s change their colours according to the kinetics that are now described.

Colour changes occur at a probabilistic rate that depends on the local density of white, or equivalently black, cells within three different length-scales: (i) only cells that have a neighbour of opposite colour may flip their colour; this is *activation at the interface.* The list of colours among the 8 neighbouring cells of $\mathbf{x}$ is denoted by $\mathbb{N}_{\mathbf{x}}$. (ii) Around each $\mathbf{x}$ we consider a concentric sphere $L_{\mathbf{x}}$ of radius r_L (in units of a cell

diameter) that is constant and common to all $\mathbf{x}$. Denote by $w_{L_\mathbf{x}}$ the fraction of white cells among all cells in $L_\mathbf{x}$, and denote by $b_{L_\mathbf{x}}$ the fraction of black cells among all cells in $L_\mathbf{x}$. Of course we have $w_{L_\mathbf{x}} + b_{L_\mathbf{x}} = 1$. The rate of flipping from black to white decreases with $w_{L_\mathbf{x}}$ and increases with $b_{L_\mathbf{x}}$; this is the condition of *long-range inhibition.* We associate with this inhibition a noise, T_L, which as $T_L \to \infty$ wipes out the long-range inhibition. (iii) Just as for (ii), around each $\mathbf{x}$ we consider a concentric sphere $S_\mathbf{x}$ of radius $r_S(< r_L)$ that is constant and common to all $\mathbf{x}$. Now the rate of flipping from black to white increases with $w_{S_\mathbf{x}}$ and decreases with $b_{S_\mathbf{x}}$; this is the condition of *short-range activation.* Again, we associate with this activation a noise, T_S, which as $T_S \to \infty$ wipes out the short-range activation.

The precise form of the reaction kinetics now follows. As a time increment Δt elapses from t to $t + \Delta t$, $U(t)$ may evolve by a colour change at one cell along the interface according to

$$
\begin{aligned}
W \in \mathbb{N}_\mathbf{x}: &\quad B \to W \quad \text{with prob.} \quad e^{\,\beta + T_S^{-1}(w-b)_{S_\mathbf{x}} - T_L^{-1}(w-b)_{L_\mathbf{x}}}\Delta t \\
B \in \mathbb{N}_\mathbf{x}: &\quad W \to B \quad \text{with prob.} \quad e^{-\beta + T_S^{-1}(b-w)_{S_\mathbf{x}} - T_L^{-1}(b-w)_{L_\mathbf{x}}}\Delta t
\end{aligned}
$$

The derivation of the mean-field equation is below. This probabilistic dynamical system is simulated by the Gillespie algorithm [1] which is exact in the sense that there is no finite time-step approximation.

2 Changing the domain size and the domain geometry

For lateral inhibition only ($T_S = \infty$, $T_L \ll 1$), striped attractors have a wavelength of $4r_L/3$ [2]; similarly for labyrinths ($T_S = T_L \ll 1$) with $r_S \ll r_L$, but the wavelength increases as r_S increases, see Figure S1a. Changing the domain size $l \times l$ over a 4-fold range appears to have no effect on the pattern's wavelength for either stripes or labyrinths, see Figure S1b. Stripes and labyrinths tend to orient perpendicularly to the domain boundary; away from the boundary, domain geometry does not appear to affect the local structure of the pattern, see Figure S1c.

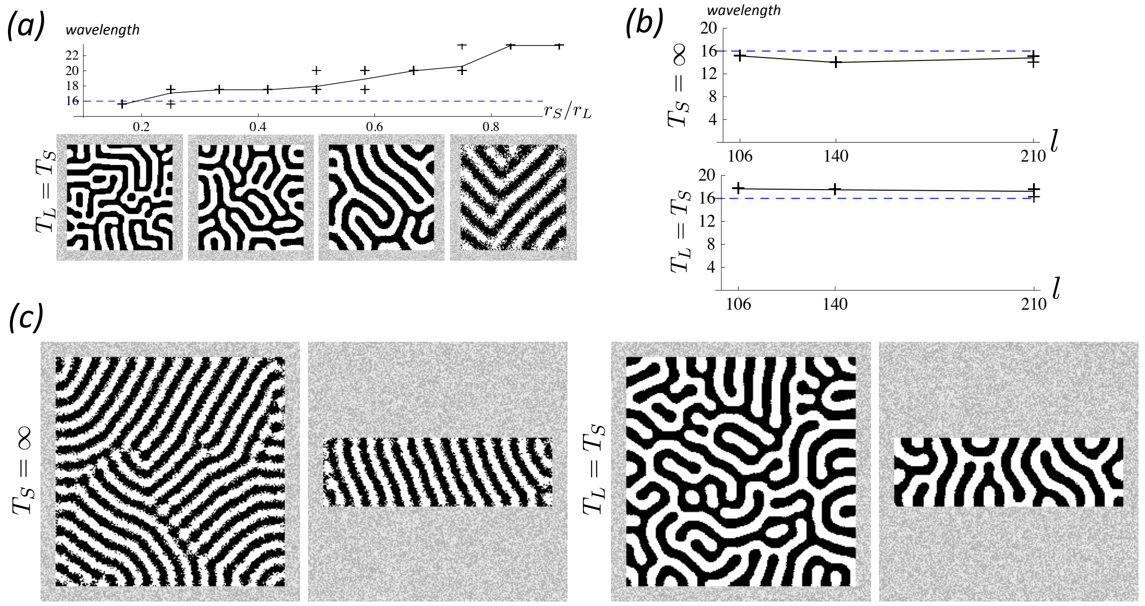

Figure S1: (a) The wavelengths of labyrinths, in units of the diameter of a lattice site, are $\approx 4r_L/3$ for $r_S \ll r_L$ (blue dashed line) then increase as r_S increases. (b) For both stripes with $r_L = 12$ (top) and labyrinths with $r_S = r_L/3 = 4$ (bottom), attractors' wavelengths vary little from $4r_L/3$ (blue dashed line) with domain length l so long as $l/r_L \gg 1$. (c) Stripes and labyrinths tend to orient perpendicularly to the domain boundary; away from the boundary, domain geometry does not appear to affect the local structure of the pattern. See Table S1 for parameter values.

3 The validity of the mean-field approximation for stationary interfaces

Time-invariant patterned attractors must have reactions $B \to W$ and $W \to B$ happening at equal rates along stationary interfaces. This gets the mean-field approximation

$$(w-b)_{L_\mathbf{x}}/T_L \; - \; (w-b)_{S_\mathbf{x}}/T_S \quad \approx \quad \beta \quad \text{for all } \mathbf{x} \text{ on the interface.} \qquad (1)$$

When $\beta = 0$, this simplifies to

$$(w-b)_{L_\mathbf{x}} \approx T_L/T_S \, \times (w-b)_{S_\mathbf{x}} \quad \text{for all } \mathbf{x} \text{ on the interface.}$$

Figure S2 demonstrates the validity of this approximation for $\beta = 0$. It shows the end state of simulations for varying T_L/T_S (left) and the corresponding scatter plots of $(w-b)_{S_\mathbf{x}}$ vs $(w-b)_{L_\mathbf{x}}$ (left-middle) evaluated at every cell on the interface (i.e. every

cell that is adjacent to an oppositely coloured cell, and so has non-zero probability of flipping its colour). The scatter plots and their density projections along orthogonal axes $t \times (-2, 1) : t \in \mathbb{R}$ (right-middle) and $t \times (1, 2) : t \in \mathbb{R}$ (right) demonstrate how the anti-correlation for $T_L/T_S \ll 1$ shifts to a positive correlation as T_L/T_S increases; the mean-field approximation predicts this qualitative trend. The negative correlation for $T_L/T_S = 0$ is due to the finite size of the sphere L.

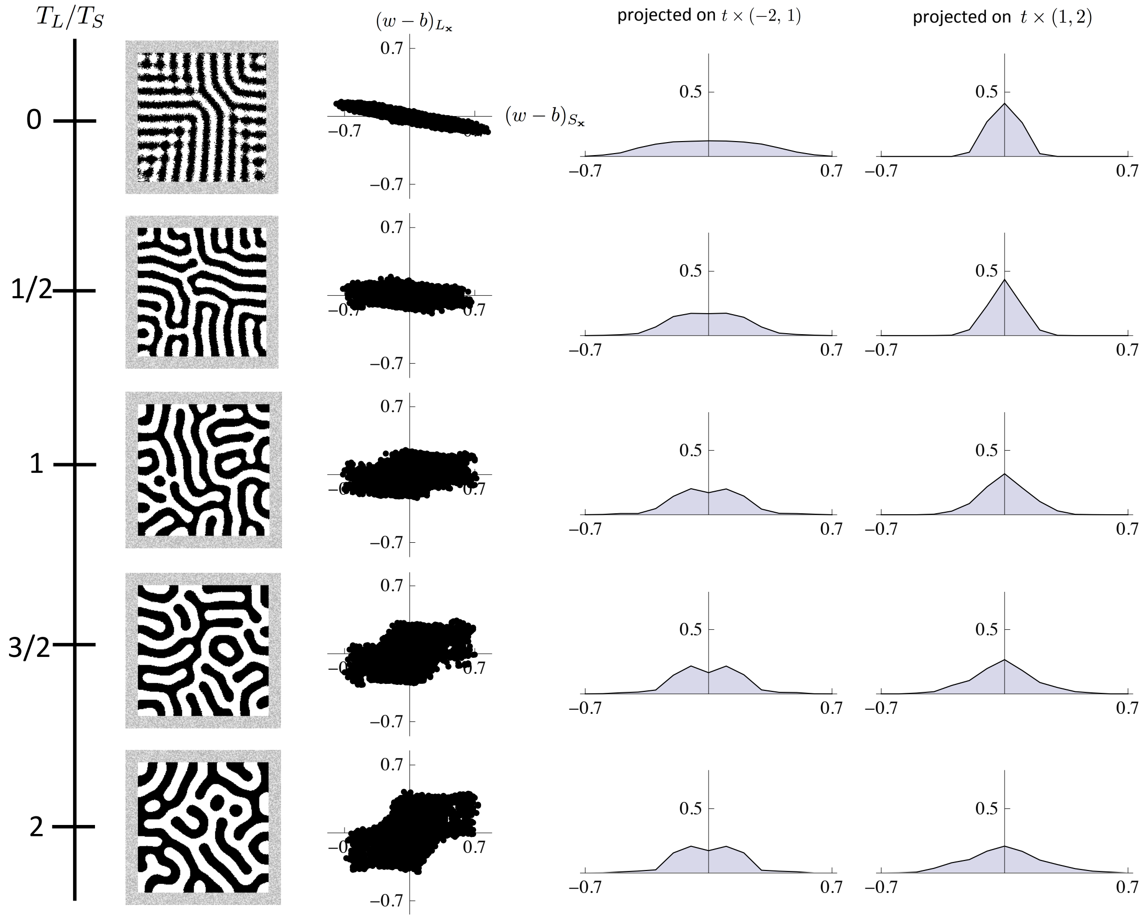

Figure S2: The end state of simulations for varying T_L/T_S (left) and the corresponding scatter plots of $(w - b)_{S_\times}$ vs $(w - b)_{L_\times}$ (left-middle) evaluated at every cell on the interface. Density projections of the scatter plots along orthogonal axes $t \times (-2, 1) : t \in \mathbb{R}$ (right-middle) and $t \times (1, 2) : t \in \mathbb{R}$ (right).

4 A justification of the form of the reaction kinetics

Consider a more general form of the reaction kinetics for two colours, but retaining only one range of lateral feedback r_J because the following analysis is straightforward to extend to an arbitrary number of ranges,

$$\begin{aligned} W \in \mathbb{N}_{\mathbf{x}} : \quad & C_{\mathbf{x}} = B \to W \quad \text{with prob.} \quad e^{\,\beta + T_J^{-1} f_{BW}((w-b)_{J_{\mathbf{x}}})}/Z \\ B \in \mathbb{N}_{\mathbf{x}} : \quad & C_{\mathbf{x}} = W \to B \quad \text{with prob.} \quad e^{-\beta + T_J^{-1} f_{WB}((b-w)_{J_{\mathbf{x}}})}/Z \end{aligned}$$

where the functions f_{WB} and f_{BW} are yet to be constrained. Let $x = (w-b)_{J_{\mathbf{x}}}$; then $-x = (b-w)_{J_{\mathbf{x}}}$. The corresponding mean-field approximation for stationary interfaces is

$$T_J^{-1}(f_{BW}(x) - f_{WB}(-x)) = -2\beta.$$

Define

$$f_o(x) = \frac{f_{BW}(x) - f_{WB}(-x)}{2} \quad \text{and} \quad f_e(x) = \frac{f_{BW}(x) + f_{WB}(-x)}{2}$$

then the mean-field approximation can be expressed as

$$T_J^{-1} f_o(x) = -\beta,$$

that is, it does not depend on $f_e(x)$.

Since in our study we have assumed that the mean-field approximation entirely determines the spatial structure of patterned attractors, we may set $f_e(x) = 0$ without excluding interesting attractors from our results. Equivalently, we consider only functions such that $f_{BW}(x) = -f_{WB}(-x)$. In particular, if $f_{WB}(x) = x + a_1x^2 + a_2x^3 + \ldots$ then $f_{BW}(x) = x - a_1x^2 + a_2x^3 - \ldots$.

5 Summary statistics

5.1 Summary statistic for 2-colour symmetry breaking transitions

For all colour symmetry breaking bifurcations ($\beta, \beta_S, \beta_L \neq 0$), the summary statistic f_b representing the corresponding transitions between patterns is the time-averaged overall fraction of white minus black cells in the $l \times l$ domain at the end of each simulation.

5.2 *Summary statistic for the transition from stationary labyrinths to labyrinthine highways*

The summary statistic f_S, which represents the bifurcation from labyrinths to labyrinthine highways, is computed as follows. Let n_x (n_y) denote the wavenumber along the horizontal (vertical) axis of an attractor simulated on a square $l \times l$ grid with corresponding wavelength l/n_x (l/n_y), (Figure S3, left column). The total power for wavelengths of magnitude $l/\sqrt{n_x^2 + n_y^2}$, calculated from the 2D discrete Fourier transform of an attractor represented as a grid of 1s and -1s (Figure S3, middle column), is plotted in Figure S3, right column; compare the top row with the bottom row. The attractor's dominant wavelength is the value of $l/\sqrt{n_x^2 + n_y^2}$ which maximizes this power; in practice, this is the wavelength of the labyrinth pattern in the absence of interwoven spots. For $T_S = T_L \ll 1$ and $\beta = 0$ as Figure 2 of the main text, the dominant wavelength does not change as σ_S varies (not shown). The summary statistic f_S is the fraction of power concentrated among wavelengths that are smaller than 1/2 of the dominant wavelength. In practice, this corresponds to the fraction of power concentrated among the short-range spots nestled within the long-range labyrinth pattern.

5.3 *Summary statistic for the transition from stationary labyrinths to gyrating labyrinths*

The summary statistic f_L, which provides a measure of the speed of movement of the interface to represent the transition from stationary to gyrating labyrinths, is computed as follows. Arbitrary values are assigned to the colours black and white so that each lattice site is associated with one of two numerical values at any given time. Then for each time $t = n \times \Delta t, n = 1, 2, \ldots$, the change in the colour configuration $\Delta_{\mathbf{x}}(t)$ at lattice site $\mathbf{x}$ since time $t - \Delta t$ is computed. By summing the absolute values of $\Delta_{\mathbf{x}}$ over the $l \times l$ domain at time t, we get the change in area ΔA swept out by the moving interface over time period Δt. In our results, Δt is chosen so that the number of colour flips between time-points is 12% of the total number of lattice sites. The sum of lattice sites on the interface l_I is also computed at each time-point. A measure for the average speed of movement of the interface at time-point t is then $\Delta A(t)/(l_I(t) \times \Delta t)$, see Figure S4. The summary statistic f_L is the increase in this time-averaged speed above its time-averaged value for the corresponding stationary labyrinth when $\sigma_L = 0$.

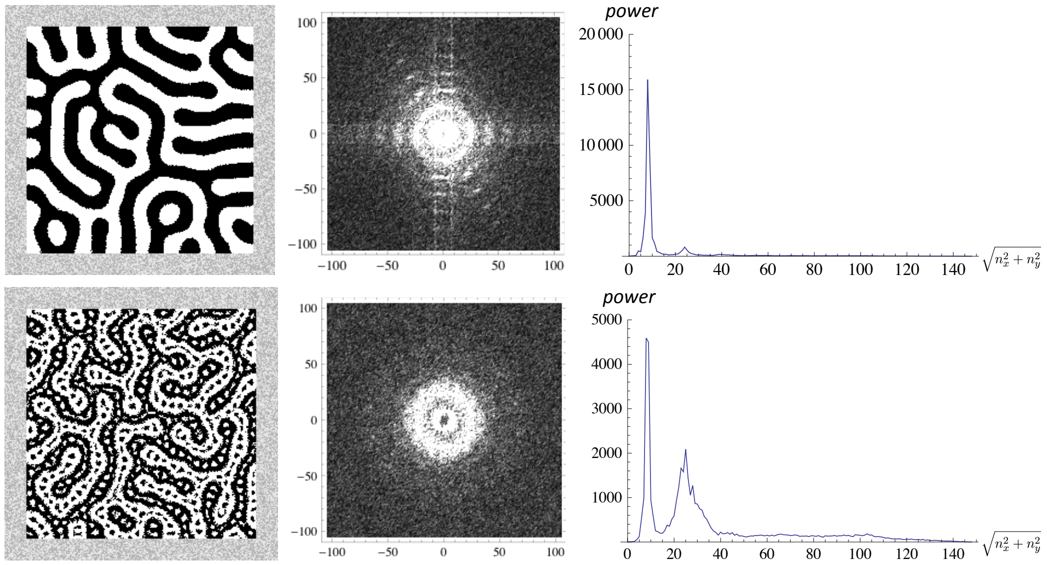

Figure S3: Attractors of diameter $l = 210$ at the 'end time' of simulations (left) and their corresponding power spectra from discrete Fourier transforms. The wavenumber along the horizontal (vertical) axis is denoted n_x (n_y). The power spectrum, which in the right plot was totalled over wavenumbers of magnitude $\sqrt{n_x^2 + n_y^2}$, becomes more concentrated among larger wavenumbers or smaller wavelengths as $|\sigma_S|$ increases beyond 1 (compare top row with bottom row).

6 The effects of boundary conditions

For 2-colour and 3-colour labyrinthine highways, 2-colour gyrating labyrinths, and 3-colour cycling spirals generated by short-range activation and cyclic symmetry breaking, the local qualitative forms of the patterning modes persist when the boundary conditions are altered so that the cells of fixed colour on the boundary all have the same colour (see Figure S5 left through to middle-right columns and Movies S11 and S12), whereas for reorganising labyrinths generated by 3-colour cyclic symmetry breaking, attractors are strongly perturbed (see Figure S5 right column and Movie S13).

7 The effects of varying noise over lateral ranges

In the main text, 2-colour patterns are invariably simulated for $T_S = T_L$. For labyrinthine highways, we varied the value of T_S relative to T_L by $\pm 25\%$ to find that the local qualitative form of the labyrinthine highway persists, albeit becoming more fuzzy with increased noise strength, see left panel Figure S6. Similarly, for gyrating labyrinths, as

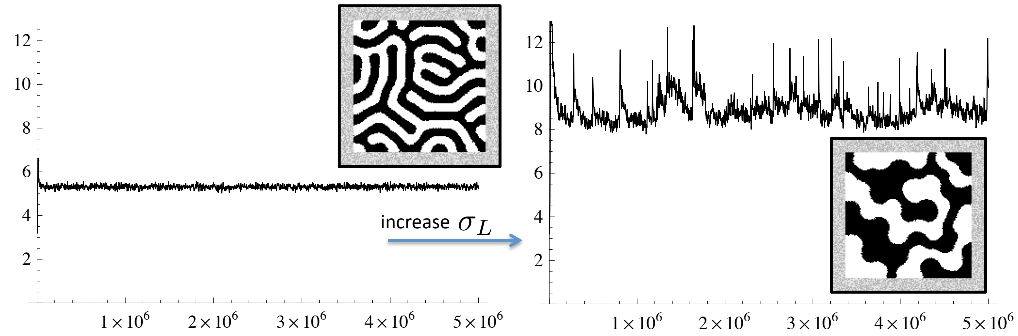

Figure S4: The time evolution of $\Delta A(t)/(l_I(t) \times \Delta t)$, a measure of the speed of movement of the interface. The time-averaged value of $\Delta A(t)/(l_I(t) \times \Delta t)$ increases as $|\sigma_L|$ increases above some threshold greater than 1 (see main text, Section 3.2).

the value of T_L is varied relative to T_S by $\pm 25\%$, the gyrations persist while their spatial reach decreases as the noise level decreases, see right panel of Figure S6 (corresponding movies not shown).

8 Mean-field equations for generalisations of the simple model of lateral activation and inhibition

Recall that the Markov chain $U(t)$ represents the colour configurations of cells that at any instant take one colour from a finite list $C_1, C_2, \ldots, C_n$ in a spatial domain Ω as they evolve in time according to reactions $C_i \to C_j$, and that $U_{\mathbf{x}}(t)$ denotes the colour of $U(t)$ at cell $\mathbf{x} \in \Omega$. We shall now attempt to derive a system of coupled mean-field equations that represent the evolution of $U(t)$ in a form akin to continuous time PDEs. In order to do this, real values must be assigned to colours, and the reaction kinetics in the model must be approximated by PDE operators. We define a collection of functions over $\mathbf{y} \in \Omega$:

$$f^{kl}(U, \mathbf{y}, t) = \begin{cases} 1 \text{ if } U_{\mathbf{y}}(t) = C_k \\ -1 \text{ if } U_{\mathbf{y}}(t) = C_l \\ 0 \text{ otherwise} \end{cases}$$

for every colour pair $k, l = 1, \ldots, n, k \neq l$. We have defined $n(n-1)$ functions, but only $n-1$ of these functions are independent since, for a given k, the subset $f^{ki}, i = 1, \ldots, n, i \neq k$, completely specifies U. The operator associated with sphere L which can

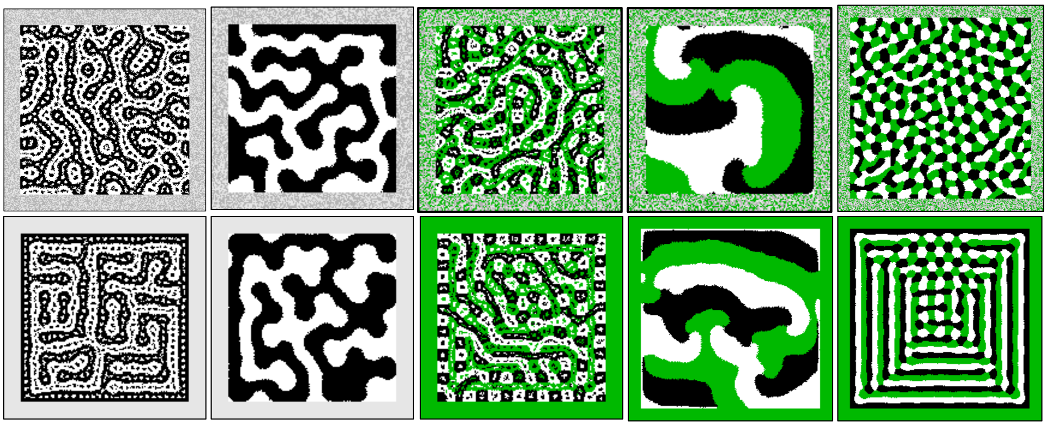

Figure S5: Altering the boundary conditions from fixed cells on the boundary equally and independently probable to to all fixed cells having a single colour (top versus bottom row) appears to have no impact on the qualitative forms of patterning modes for labyrinthine highways, gyrating labyrinths, and cyclic swirls (left through to middle-right column and Movies S11 and S12), while the impact is stronger for reorganising labyrinths (right column and Movie S13) that are generated by 3-colour short-range activation and long-range inhibition plus cyclic symmetry breaking.

be considered a discrete analog of the Laplacian is defined over $\mathbf{y} \in \Omega$ to be:

$$\triangle_L f(U, \mathbf{y}, t) = \sum_{\mathbf{w} \in L_{\mathbf{y}}} f(U, \mathbf{w}, t)/N_L - f(U, \mathbf{y}, t)$$

where N_L is the total number of cells in sphere L. Similarly for sphere S. Then, according to the model's definition

$$(\triangle_L + 1) f^{kl}(U, \mathbf{y}, t) = (c_k - c_l)_{L_{\mathbf{y}}}$$

and along the interface kl, since $f^{kl}(U, \mathbf{y}, t)$ is -1 on one side of the interface and $+1$ on the other, we may approximate $f^{kl}(U, \mathbf{y}, t) \approx 0$, so

$$\triangle_L f^{kl}(U, \mathbf{y}, t) = (c_k - c_l)_{L_{\mathbf{y}}} \text{ along the interface } kl.$$

This simple form justifies our choice of functions $\{f^{kl}\}$. The following sets of functions will also be important in what follows below. Define the set $\{I^i\}, i = 1, \ldots, n$ over $\mathbf{y} \in \Omega$

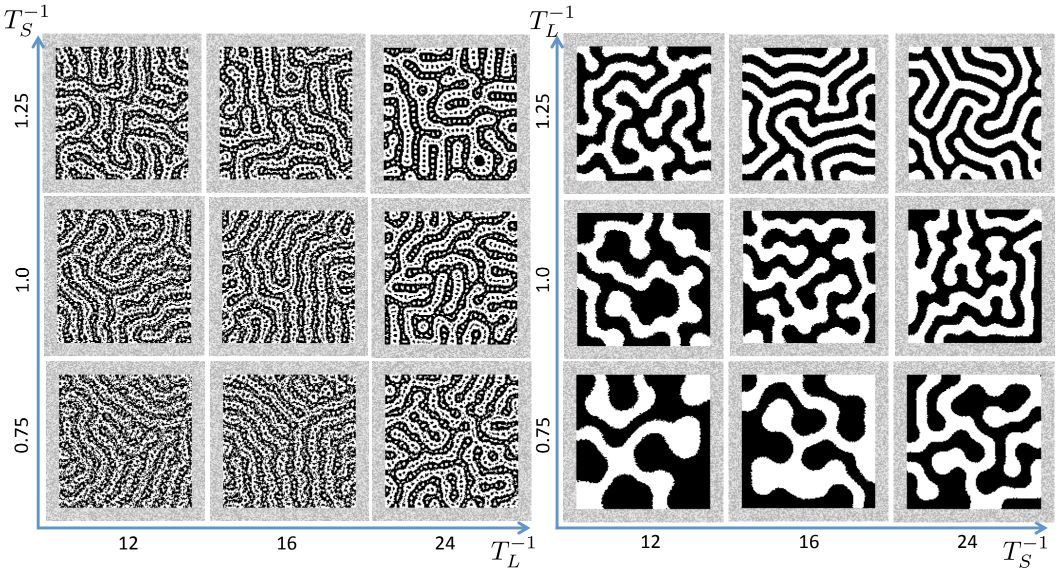

Figure S6: Altering noise on the short-range (T_S) and long-range (T_L) by ±25% has little affect on the qualitative features of labyrinthine highways (left panel) and gyrating labyrinths (right panel).

to be

$$I^i(U, \mathbf{y}, t) = \begin{cases} 1 & \text{if} \quad U_{\mathbf{y}} = C_i \\ 0 & \text{otherwise,} \end{cases}$$

which can be expressed in terms of $\{f^{ij}\}$ s as

$$I^i = \frac{\sum_{j:j\neq i} f^{ij} + 1}{n}. \tag{2}$$

While the functions

$$N^i(U, \mathbf{y}, t) = \begin{cases} 1 & \text{if a cell neighbouring } \mathbf{y} \text{ has colour } C_i \\ 0 & \text{otherwise,} \end{cases}$$

are given by

$$N^i(U, \mathbf{y}, t) = 1 - \prod_{\mathbf{w}\in\mathbb{N}_{\mathbf{y}}} \Big(1 - \prod_{j:j\neq i} (1 - I^j(U, \mathbf{w}, t))\Big) \tag{3}$$

where $\mathbb{N}_{\mathbf{y}}$ is the set of cells neighbouring $\mathbf{y}$. Note that, according to the model's definition, for a given configuration U at time t the rate of the colour flip $C^i_{\mathbf{x}} \to C^j_{\mathbf{x}}$ is non-zero

if and only if $(I^i \times N^j)(U,\mathbf{x},t) = 1$.

Some new notation is introduced in order to simplify the expression for the Kolmogorov forward equation, an evolution equation for the probability distribution of U, which must necessarily be written down to derive the mean-field equations. For each $\mathbf{x} \in \Omega$ and each colour pair (i,j) $i \neq j$, define a flip operator $F^{ij}_{\mathbf{x}}$, which, if cell $\mathbf{x}$ has colour C_i, flips it to colour C_j, and otherwise $F^{ij}_{\mathbf{x}}$ does nothing:

$$\left(F^{ij}_{\mathbf{x}}(U)\right)_{\mathbf{y}} = U_{\mathbf{y}} \text{ for all } \mathbf{y} \neq \mathbf{x} \in \Omega, \quad \left(F^{ij}_{\mathbf{x}}(U)\right)_{\mathbf{x}} = \begin{cases} C_j & \text{if } \quad U_{\mathbf{x}} = C_i \\ U_{\mathbf{x}} & \text{otherwise.} \end{cases}$$

Note that $F^{ji}_{\mathbf{x}}(F^{ij}_{\mathbf{x}}(\cdot)) = I(\cdot)$, the identity map.

The rate of the reaction $U_{\mathbf{x}} = C_i \to C_j$ is represented below by $r_{U\,F^{ij}_{\mathbf{x}}(U)}$ which equals zero if $U_{\mathbf{x}} \neq C_i$. The Kolmogorov forward equation (see e.g. [3]) is

$$\frac{d}{dt}P\{\underline{u} = U(t)\} = \sum_{\mathbf{x}} \sum_{(i,j):i\neq j} r_{F^{ij}_{\mathbf{x}}(U)\,U} P\{\underline{u} = F^{ij}_{\mathbf{x}}(U)\} - \sum_{\mathbf{x}} \sum_{(i,j):i\neq j} r_{U\,F^{ij}_{\mathbf{x}}(U)} P\{\underline{u} = U\}$$

where $P\{\cdot\}$ is the probability of $\{\cdot\}$ conditioned upon the initial state of U. So, for any real-valued function $f(U)$, we have

$$\begin{aligned} \frac{d}{dt}\mathbb{E}[f(U)] &= \frac{d}{dt}\sum_{U} f(U)P\{\underline{u} = U(t)\} = \sum_{U} f(U)\,\frac{d}{dt}P\{\underline{u} = U(t)\} \\ &= \sum_{U} f(U) \sum_{\mathbf{x},(i,j):i\neq j} \left(r_{F^{ij}_{\mathbf{x}}(U)\,U} P\{\underline{u} = F^{ij}_{\mathbf{x}}(U)\} - r_{U\,F^{ij}_{\mathbf{x}}(U)} P\{\underline{u} = U\}\right) \\ &= \sum_{U'} \sum_{\mathbf{x},(j,i):j\neq i} f(U')\,r_{F^{ji}_{\mathbf{x}}(U')\,U'} P\{\underline{u} = F^{ji}_{\mathbf{x}}(U')\} - \sum_{U} \sum_{\mathbf{x},(i,j):i\neq j} f(U)\,r_{U\,F^{ij}_{\mathbf{x}}(U)} P\{\underline{u} = U\}. \end{aligned} \tag{4}$$

For each triplet $(U, \mathbf{x}, (i,j)), i \neq j$, there is a corresponding unique triplet $(U', \mathbf{x}, (j,i)), j \neq i$ such that $F^{ji}_{\mathbf{x}}(U') = U$ and $F^{ij}_{\mathbf{x}}(U) = U'$. Consequently $r_{F^{ji}_{\mathbf{x}}(U')\,U'} = r_{U\,F^{ij}_{\mathbf{x}}(U)}$ and we see that there is a pairwise cancellation for every term such that $f(F^{ij}_{\mathbf{x}}(U)) = f(U), i \neq j$. Then (4) simplifies to

$$\frac{d}{dt}\mathbb{E}[f(U)] = \sum_{U} \sum_{\mathbf{x},(i,j):i\neq j} \left(f(F^{ij}_{\mathbf{x}}(U)) - f(U)\right) r_{U\,F^{ij}_{\mathbf{x}}(U)}\, P\{\underline{u} = U\}, \tag{5}$$

where $\mathbb{E}[f(U)]$ is the expectation of $f(U)$ conditioned upon the initial state of U.

Substituting into (5) the function f^{kl} gets

$$\begin{aligned}\frac{d}{dt}\,\mathbb{E}[f^{kl}(U,\mathbf{y},t)] &= \sum_U \sum_{(i,j),i\neq j} \Big(f^{kl}(F^{ij}_{\mathbf{y}}(U),\mathbf{y},t) - f^{kl}(U,\mathbf{y},t)\Big)\, r_{U\,F^{ij}_{\mathbf{y}}(U)}\, P\{\underline{u}=U\} \\ &= \sum_U \Big(\sum_{i\neq k}(r_{U\,F^{ik}_{\mathbf{y}}(U)} - r_{U\,F^{ki}_{\mathbf{y}}(U)}) - \sum_{i\neq l}(r_{U\,F^{il}_{\mathbf{y}}(U)} - r_{U\,F^{li}_{\mathbf{y}}(U)})\Big)\, P\{\underline{u}=U\} \\ &= \mathbb{E}\Big[\sum_{i\neq k}(r_{U\,F^{ik}_{\mathbf{y}}(U)} - r_{U\,F^{ki}_{\mathbf{y}}(U)}) - \sum_{i\neq l}(r_{U\,F^{il}_{\mathbf{y}}(U)} - r_{U\,F^{li}_{\mathbf{y}}(U)})\Big]. \qquad (6)\end{aligned}$$

When there are only 2 colours, C_k and C_l, (6) simplifies to

$$\frac{d}{dt}\,\mathbb{E}[f^{kl}(U,\mathbf{y},t)] \;=\; 2\mathbb{E}\Big[r_{U\,F^{lk}_{\mathbf{y}}(U)} - r_{U\,F^{kl}_{\mathbf{y}}(U)}\Big].$$

The final challenge is to express $r_{U\,F^{ij}_{\mathbf{x}}(U)}$ in terms of the functions $\{f^{kl}\}$ and approximations to familiar operators such as the Laplacian; along with assumptions such as $\mathbb{E}[x^i] = \mathbb{E}[x]^i$, this would provide a deterministic continuum model that could be simulated as a PDE. For each model that we have studied, the reaction rates take the form :

$$r_{U\,F^{ij}_{\mathbf{x}}(U)} = I^i\, N^j\, e^{g_{ij}(\triangle_S f^{ij}, \triangle_L f^{ij}, \triangle_S f^{jk}, \triangle_L f^{jk}, \ldots)}(U,\mathbf{x},t)$$

where each g_{ij} depends on the model's definition. From (2), I^i is simply a linear sum of a subset of the functions $\{f^{kl}\}$, but the expression for N^j from (3) is more complicated and we cannot see how to write N^j in terms of familiar operators acting on $\{f^{kl}\}$: the assumption of activation at the interface makes the mean-field equations challenging to derive. A second complication is due to the two lengthscales r_S and r_L which, according to our definition, result in two distinct discrete analogs to the Laplacian, $\triangle_S$ and $\triangle_L$. If the model featured only a single lengthscale r_L (this is the case for lateral inhibition only) then $\triangle_L$ corresponds to the dimensionless Laplacian operator $D/k\nabla^2$.

In future these two difficulties may be resolved, in which case it would be interesting to compare the dynamics of our discrete and probabilistic models with the corresponding continuous and deterministic models.

9 Supplementary movie captions

All movies can be found at `https://www.repository.cam.ac.uk/handle/1810/253075`.

Movie S1. Two colour symmetric lateral inhibition produces stationary striped attractors.

Movie S2. Two colour symmetric short-range lateral activation and long-range lateral inhibition produces stationary labyrinths.

Movie S3. Colour symmetric short-range activation with competing nonlinear short-range inhibition and long-range inhibition produces labyrinthine highways; the labyrinths are near stationary.

Movie S4. Colour symmetry break for short-range activation with competing nonlinear short-range inhibition and long-range inhibition. This produces near stationary Kagome lattices for $r_S/r_L = 1/2$.

Movie S5. Nonlinear colour symmetry break for short-range activation with competing nonlinear short-range inhibition and long-range inhibition. This produces near stationary train tracks.

Movie S6. Colour symmetric short-range activation and long-range inhibition with competing nonlinear long-range activation. This produces non-stationary continually gyrating labyrinths.

Movie S7. Cyclic symmetry break for three colour lateral inhibition produces travelling tri-stripes.

Movie S8. Cyclic symmetry break for three colour lateral activation produces cyclic spirals.

Movies S9 and S10. Cyclic symmetry break for three colour short-range activation and long-range inhibition produces continually reorganising labyrinths (long-range cycling–S9) and cycling checkerboards (short-range cycling–S10).

Movie S11. Colour symmetric short-range activation and long-range inhibition with competing nonlinear long-range activation with all white cells forming the boundary condition (compare with Movie S6).

Movie S12. Cyclic symmetry break for three colour lateral activation produces cyclic spirals with all green cells forming the boundary condition (compare with Movie S8).

Movie S13. Colour symmetric short-range activation and long-range inhibition with competing nonlinear long-range activation with all white cells forming the boundary condition (compare with Movie S9).

Table S1: Parameters for figures. The 'end time' is the number of transitions per simulation. n is the number of instances simulated for generating statistics. 'V' stands for 'varying' in the corresponding figure or plot.

Figure	l	β	$(r_S, T_S^{-1}, \beta_S, \sigma_S, \gamma_S)$	$(r_L, T_L^{-1}, \beta_L, \sigma_L, \gamma_L)$	end time	n
S1a)	140	0	(V, 16,–,–,–)	(12, 16,–,–,–)	2×10^6	5
S1b) top row	V	0	(–, –,–,–,–)	(12, 16,–,–,–)	2×10^6	5
S1b) bottom row	V	0	(4, 16,–,–,–)	(12, 16,–,–,–)	2×10^6	5
S1c)	210	0	(V, V,–,–,–)	(12, 16,–,–,–)	2×10^6	–
S2)	210	0	(6, V,–,–,–)	(18, 16, –, –,–)	3×10^6	–
S3) top row	210	0	(6, 16, 0, 0,–)	(18, 16, 0, 0,–)	5×10^6	–
S3) bottom row	210	0	(6, 16, 0, 2.5,–)	(18, 16, 0, 0,–)	5×10^6	–
S4) left	140	0	(6, 16, 0, 0,–)	(12, 16, 0, 0,–)	5×10^6	–
S4) right	140	0	(6, 16, 0, 0,–)	(12, 16, 0, 3,–)	5×10^6	–
S5) left	140	0	(4, 16, 0, 2,–)	(12, 16, 0, 0,–)	3×10^6	–
S5) middle-left	140	0	(6, 16, 0, 0,–)	(12, 16, 0, 2.3,–)	3×10^6	–
S5) middle	140	0	(4, 16, 0, 2,–)	(12, 16, 0, 0,–)	3×10^6	–
S5) middle-right	210	0	(4, 16, 0, 0, 1)	(–, –, –, –, –)	3×10^6	–
S5) right	210	0	(4, 16, 0, 0, 0)	(12, 16, 0, 0, 2)	3×10^6	–

References

[1] Gillespie DT (1976) General method for numerically simulating the stochastic time evolution of coupled chemical reactions. Journal of Computational Physics 22: 403–434.

[2] Willis L, Cox EJ, Duke T (2013) A simple probabilistic model of submicroscopic diatom morphogenesis. Journal of the Royal Society Interface 10: 20130067.

[3] Karlin S, Taylor HM (1975) A First Course in Stochastic Processes. Academic Press.

Table S2: Parameters for supplementary movies accompanying each figure in the main text. The 'end time' is the number of transitions per simulation.

Movie	l	β	$(r_S,\, T_S^{-1},\, \beta_S,\, \sigma_S,\, \gamma_S)$	$(r_L,\, T_L^{-1},\, \beta_L,\, \sigma_L,\, \gamma_L)$	end time
S1	210	0	(–, –, –,–,–)	(18, 16, –,–,–)	3×10^6
S2	210	0	(6, 16, –,–,–)	(18, 16, –,–,–)	3×10^6
S3	210	0	(6, 16, 0, 2,–)	(18, 16, –,–,–)	2×10^6
S4	210	2	(6, 16, 0, 2,–)	(12, 16, –,–,–)	3×10^6
S5	210	0	(6, 16, 6, 2,–)	(18, 16, –,–,–)	3×10^6
S6	210	0	(9, 16, –, –,–)	(18, 16, 0, 2,–)	5×10^6
S7	140	–	(–, –, –, –,–)	(12, 16, –, –,1)	3×10^6
S8	210	–	(4, 16, –, –,1)	(–, –, –, –,–)	3×10^6
S9	140	–	(4, 16, –, –,0)	(12, 16, –, –, 2)	3×10^6
S10	210	–	(6, 16, –, –,0.5)	(18, 16, –, –,0)	3×10^6
S11	140	0	(6, 16, –, –,–)	(12, 16, 0, 2.3,–)	3×10^6
S12	210	–	(4, 16, –, –,1)	(–, –, –, –,–)	3×10^6
S13	210	–	(4, 16, –, –,0)	(12, 16, –, –, 2)	3×10^6
